# Frailty Prevalence and Association with Health-Related Quality of Life Impairment among Rural Community-Dwelling Older Adults in Vietnam

**DOI:** 10.3390/ijerph16203869

**Published:** 2019-10-12

**Authors:** Anh Trung Nguyen, Long Hoang Nguyen, Thanh Xuan Nguyen, Thu Thi Hoai Nguyen, Huong Thi Thu Nguyen, Tam Ngoc Nguyen, Hai Quang Pham, Bach Xuan Tran, Carl A. Latkin, Cyrus S. H. Ho, Roger C. M. Ho, Thang Pham, Huyen Thi Thanh Vu

**Affiliations:** 1 Scientific Research Department, National Geriatric Hospital, Hanoi 100000, Vietnam; xuanthanh1901vlk@gmail.com (T.X.N.); nththu.bvlk@gmail.com (T.T.H.N.); thuhuonglk@hmu.edu.vn (H.T.T.N.); ngoctamyhn@gmail.com (T.N.N.); phamthang@hmu.edu.vn (T.P.); vuthanhhuyen11@hmu.edu.vn (H.T.T.V.); 2 Department of Gerontology, Hanoi Medical University, Hanoi 100000, Vietnam; 3 Center of Excellence in Behavioral Medicine, Nguyen Tat Thanh University, Ho Chi Minh City 700000, Vietnam; longnh.ph@gmail.com (L.H.N.); pcmrhcm@nus.edu.sg (R.C.M.H.); 4 Dinh Tien Hoang Institute of Medicine, Hanoi 100000, Vietnam; 5 Institute for Global Health Innovations, Duy Tan University, Da Nang 550000, Vietnam; quanghai23hmu@gmail.com; 6 Institute for Preventive Medicine and Public Health, Hanoi Medical University, Hanoi 100000, Vietnam; bach.jhu@gmail.com; 7 Bloomberg School of Public Health, Johns Hopkins University, Baltimore, MD 21205, USA; carl.latkin@jhu.edu; 8 Department of Psychological Medicine, National University Hospital, Singapore 119074, Singapore; cyrushosh@gmail.com; 9 Institute for Health Innovation and Technology (iHealthtech), National University of Singapore, Singapore 119077, Singapore; 10 Department of Psychological Medicine, Yong Loo Lin School of Medicine, National University of Singapore, Singapore 119228, Singapore

**Keywords:** frailty, health-related quality of life, older adult, Vietnam

## Abstract

Measuring health-related quality of life (HRQOL) is critical to evaluate the burden of frailty in the older population.This study explored the prevalence of frailty among Vietnamese older people in rural communities, determined the factors associated with frailty, and examined the differences in HRQOL between non-frail, pre-frail, and frail people. A cross-sectional study was conducted on older adults (≥60 years old) residing in Soc Son district, northern Vietnam. Non-frailty, pre-frailty, and frailty conditions were evaluated using Fried’s frailty criteria. The EuroQol-5 Dimensions-5 Levels(EQ-5D-5L) instrument was employed to measure HRQOL. Socioeconomic, behavioral, health status, and healthcare utilization characteristics were collected as covariates. Among 523 older adults, 65.6% were pre-frail, and 21.7% were frail. The mean EQ-5D-5L indexes of the non-frailty, pre-frailty, and frailty groups were 0.70 (SD = 0.18), 0.70 (SD = 0.19), and 0.58 (SD = 0.20), respectively. The differences were found between non-frailty and frailty groups (*p* < 0.01), as well as the pre-frailty and frailty groups (*p*<0.01). After adjusting for covariates, the estimated mean difference in the HRQOL between the non-frailty and frailty groups was −0.10 (95%CI= −0.17; −0.02) (R^2^ = 45.2%), showing a 10% reduction of the maximum EQ-5D-5L index.This study emphasized the high prevalence of frailty among older adults in the rural communities of Vietnam. Frailty was found to be associated with a small reduction of HRQOL in this population.

## 1. Introduction

Frailty is an important age-related health issue that is characterized by the significant reduction of physiological reserves and the increasing vulnerability to stressors [1]. Prior literature underlines that frailty phenotype (from pre-frailty to frailty) is a critical predictor for severe adverse health conditions such as cardiovascular diseases [2], depression [3], hospitalization, loss of basic daily activities, falls, fractures, or premature mortality [4,5]. The risk of frailty increases with age due to the gradual decrease of functional capacity and increase of functional dependence; therefore, it is particularly common in older adults [6,7]. Moreover, factors that have been recognized as significant risk factors for the occurrence of frailty include being female, single marital status, a lack of social support, a higher rate of comorbidities, disability, and functional limitation [8,9,10]. Nonetheless, there is no consensus of the frailty rate across countries. In developed countries, a systematic review in 2012 indicated that the frailty prevalence among older adults in the community was 10.7% [4]. Meanwhile, in the low and middle-income countries, the frailty prevalence in this population ranged from 3.9% in China to 51.4% in Cuba, and the pre-frailty rate varied from 13.4% in Tanzania to 71.6% in Brazil [6]. Older dwellings living in rural areas were more susceptible to frailty than urban ones [11,12], which might be due to the limited accessibility of healthcare services and resources [13]. Therefore, since population aging is an undeniable phenomenon around the globe [14], more contextualized evidence about the prevalence of frailty and its effects on health outcomes in older people, particularly in rural areas, are critically important for developing strategies to cope with this emergent issue.

Currently, there are many methods used to measure frailty, of which Fried phenotype assessment is the most common [15] thanks to its good validity, reliability, and responsiveness to the change of frailty level in the trial [1,16,17,18,19]. This instrument used a collection of five specific symptoms including weight loss, exhaustion, low physical activity, slowness, and weakness to evaluate the frailty level of each person [1]. Another emerging approach to assess frailty is a frailty index (FI), which is calculated from the accumulation of health deficits [20]. This method requires a remarkable number of items to fully assess the deficits of each individual. The utility of each method has been debated. For instance, several studies showed that FI was more accurate for mortality prediction compared to Fried’s phenotype method [18,21], while other work found that these two methods were comparable [22,23]. However, Shi et al. argued that in the community setting, FI was based mainly on self-reported data, which increased the risk of bias [24]. Moreover, the phenotype approach is short and simple, requiring a far less amount of information compared to the FI approach [25,26]. Collectively, it is suitable to use in the community to screen the frailty condition among older people in the community.

In literature, health-related quality of life (HRQOL) has been increasingly considered an important indicator for measuring the burden of frailty on the community’s health [27,28,29]. HRQOL is defined as “How well a person functions in their life and his or her perceived well-being in physical, mental, and social domains of health’’ [30]. HRQOL is associated with an increased risk of major chronic diseases such as arthritis, as well as heart and lung diseases [31]. Evidence shows that rural older people had lower HRQOL than those living in urban areas [32,33]. Among older people, poor HRQOL is a good predictor for mortality [34]. Therefore, HRQOL becomes a favorable indicator to measure the impact of illnesses or diseases on health outcome. Previous systematic reviews reveal a strong connection between frailty and HRQOL impairment [28,29]. However, most of the studies pooled in these reviews were from high-income countries, while limited studies were conducted in low and middle-income countries such as Brazil [35] and China [36]. Therefore, more contextualized evidence about the effect of frailty on HRQOL is necessary to understand the burden of frailty on older population in developing countries.

In community aged care, the EuroQol-5 Dimensions (EQ-5D) is a predominant instrument that is utilized to measure the HRQOL of older people [37]. This is a short and simple generic preference-based measure for evaluating HRQOL, which covers briefly five dimensions including mobility, self-care, usual activity, pain/discomfort, and anxiety/depression, causing a minimal response burden to participants, especially older people, compared to other HRQOL instruments [37]. Moreover, this instrument can offer a single “utility” index for different health states, which is important component for computing the Quality-Adjusted Life Years (QALYs)—a vital outcome for economic evaluations [38]. A new version of EQ-5D called EuroQOL-5 Dimensions-5 Levels (EQ-5D-5L) has been released to reduce the ceiling effect and increase the applicability of this instrument in measuring the effects of interventions [39,40]. Thus, it is necessary to acquire evidence about the difference in HRQOL using the EQ-5D-5L between frail and non-frail older adults, which can serve as a baseline characteristic for further interventions to improve this problem in the community.

Population aging is a substantial phenomenon in Vietnam given a rapidly increased proportion of older people from 8.9% in 2009 to 30% in 2050 [14,41]. However, studies about frailty among Vietnamese older adults in the community are lacking. One prior study in the hospital revealed that the frailty rate among hospitalized older patients was 35.4% [42]. Therefore, this study was conducted to explore the prevalence of frailty among Vietnamese older people in rural communities, determine the associated factors with frailty, as well as examine the differences in HRQOL between non-frail, pre-frail, and frail people.

## 2. Materials and Methods

### 2.1. Participants and Study Procedure

The Institutional Review Board of the National Geriatric Hospital approved this study protocol (Reference: No 35; 16/01/2017). A cross-sectional study was performed in Soc Son district, Hanoi—a typical rural community in northern Vietnam based on the classification of the Vietnam Government [43]. Older individuals aged 60 years old or more who have resided in this district at least 6 months, and agreed to participate and give written informed consent were invited into the study. Older dwellings were excluded if they had 1) severe illnesses or 2) cognitive disorders that might affect their capacity to answer the questions. First, 530 older adults were randomly selected from the sample frame of approximately 33,000 eligible people in the district by using STATA software (Stata Corp. LP, College Station, TX, USA). The sample frame was obtained from the district’s local authority. Second, the participants’ houses were visited, and they were invited to participate in the study after the brief introduction of local health workers. A total of 523 (98.7%) older people accepted the invitation.

### 2.2. Data Collection

Data collection was conducted from February to April 2017 via face-to-face interviews. A structured questionnaire was developed and piloted in 10 people to ensure the language and logical order. Interviewers included undergraduate medical students at the Hanoi Medical University, who received two-day intensive training to assure the quality of collected data. Information of interest included socioeconomic and behavioral characteristics (age, gender, occupation, marital status, caregiver, alcohol and tobacco consumption in the last five years). We also asked participants to report health status and health service utilization, namely history of morbidity (such as stroke, transient ischemic attack, diabetes mellitus, chronic lung disease, Parkinson’s, arthritis, and hypertension), cognitive impairment (using the Mini Cog instrument [44]), history of falls in the last 12 month, polypharmacy (≥5 drugs), and using inpatient/outpatient/regular health examination services in the last 12 months.

Frailty phenotype: in this study, we adapted five criteria that were proposed by Fried et al. [1], including:

(a) Weight loss: ≥5% or 4.5 kg compared to weight in the last 12 months.
(b) Exhaustion: Two questions in the Centre for Epidemiologic Studies Depression Scale were used, including “I felt that everything I did was an effort last week”, and “I couldn’t get going last week.” People had exhaustion if they answered “frequently” or “always” in either of the two questions.
(c) Low physical activity: Participants responding “I rarely or never do any physical activities” were classified into the “low physical activity” group.
(d) Slowness: ≥5 seconds for 4-meter gait speed test [45].
(e) Weakness: We used a dynamometer (Jamar TM Hidraulic Hand Dynamometer 5030 J1, Fred Sammons, Inc., Burr Ridge, IL, USA) to measure grip strength in the dominant hand. People had weakness if they had the lowest quintile of grip strength after adjusting for sex and body mass index. The cut-off points were: (a) in men: BMI <18.50: grip strength <6.8 kg; BMI 18.50–24.99: grip strength <12.0 kg; BMI ≥25: grip strength <12.6 kg; (b): in women: BMI <18.50: grip strength <4.0 kg; BMI 18.50–24.99: grip strength <6.0 kg; BMI ≥25: grip strength <9.8 kg [42].

Participants were categorized into three groups: non-frailty (score 0), pre-frailty (score 1–2), and frailty (score 3–5) [1]. Those belonged to the first two categories were grouped into the “non-frailty” category. This measure and its cut-off point had been used previously among older patients in a Vietnamese hospital setting [42].

Health-related quality of life: The EuroQol-5 Dimensions-5 Levels (EQ-5D-5L), a generic preference-based instrument, was used to evaluate HRQOL in five dimensions: (1) Mobility; (2) Self-care; (3) Usual activities; (4) Pain/discomfort, and (5) Anxiety/depression. There are five options of response for each dimension: from “No problem” (code 1) to “Extreme problems” (code 5) [46]. The combination of answers offers 3125 possible health states (from 11111, full health, to 55555, worst health) [46], which are represented by 3125 single “utility” index (from −0.451 (for 55555) to 1.000 (for 11111)) using the Thailand cross-walk value set [46]. We grouped participants reporting “No problem” to the “No problem” category, and grouped other people to the “Having problems” category [13].

### 2.3. Data Analysis

Data were analyzed by using STATA 14.0 software (Stata Corp. LP, College Station, TX, USA). The chi-squared test was utilized to examine the difference in socioeconomic characteristics, behavioral characteristics, health status, and health service utilization between non-frailty, pre-frailty, and frailty groups. We employed the Kruskal–Wallis to test the difference in age and EQ-5D-5L index among these three groups, because these data had non-normal distribution (Skewness–Kurtosis test). Dunn’s test was used to show the specific group having a significantly different EQ-5D-5L index compared to the other groups.

Multivariate logistic regression was used to determine associated factors with frailty status. A stepwise forward selection strategy was combined with the logistic regression model to provide a final adjusted reduced model. A *p*-value of 0.2 was employed as a threshold of variable selection. This threshold could prevent the removal of variables that are potentially related to frailty status. An adjusted odds ratio (OR) was presented along with the *p*-value and a 95% confident interval. For the EQ-5D-5L index, univariate (model 1) and multivariate Tobit regression (model 2–7) models were employed to examine the difference in HRQOL between non-frailty and pre-frailty, and non-frailty and frailty groups. Goodness of fit (R^2^) was explored. A *p*-value of less than 0.05 was recognized as statistically significant.

## 3. Results

Of the 523 older adults participating in the study, 65.6% were pre-frail, and 21.7% were frail. The mean age of the non-frailty, pre-frailty, and frailty groups were 67.4 (SD = 4.9) years, 72.6 (SD = 7.7) years, and 76.7 (SD = 9.2) years old, respectively. Most of the respondents were female (69.9%), with a non-significant difference among groups (*p* = 0.184). Also, no difference was found in smoking status and alcohol use among groups (*p* > 0.05). Otherwise, there were significant differences in age, marital status, occupation, and caregiver in the prevalence of non-frailty, pre-frailty, and frailty groups (*p* < 0.05), (Table 1).

Table 2 reveals that the proportion of frail participants suffering from diabetes mellitus (10.8%), hypertension (44.1%), fall in the last 12 months (30.6%), cognitive impairment (80.2%), and polypharmacy (21.6%) were higher than those of the non-frail (1.5%, 20.0%, 18.5%, 40.0%, and 4.6%, respectively) and pre-frail groups (3.3%, 31.0%, 18.8%, 67.0%, and 13.4%, respectively). These differences were statistically significant (*p* < 0.05).

Table 3 presents the result of a stepwise multivariate logistic regression. Increasing one year of age would increase the chance of suffering frailty by 5% (OR = 1.05, *p* < 0.01). People who were widowed had a 1.73 times higher risk of experiencing frailty compared to those having a spouse/partner (OR = 1.73, *p* = 0.03). Farmers were less likely to report frailty than retired individuals (OR = 0.54, *p* = 0.03). Older adults that had cognitive impairment (OR = 1.88, *p* = 0.02) and had fallen in the last 12 months (OR = 1.91, *p* = 0.02) were more likely to have frailty. Experiencing one (OR = 2.33, *p* < 0.01) or two or more (OR = 1.97, *p* = 0.03) comorbidities was associated with a higher likelihood of having frailty.

Table 4 shows the distribution of people having problems in each dimension, as well as the EQ-5D-5L index across three groups. The mean EQ-5D-5L indexes in the non-frailty, pre-frailty, and frailty groups were 0.70 (SD = 0.18), 0.70 (SD = 0.19), and 0.58 (SD = 0.20), respectively. The differences were found between non-frailty and frailty (*p* < 0.01), as well as the pre-frailty and frailty groups (*p* < 0.01).In all dimensions, frail people reported a significantly higher proportion of problems (e.g., mobility (74.8%), self-care (42.3%), usual activity (64.9%), pain/discomfort (81.1%), and anxiety/depression (52.3%)) than the other two groups (*p* < 0.05).

Table 5 illustrates several Tobit regressions to identify the association between frailty status and EQ-5D-5L index. In the unadjusted model, the estimated mean difference in the EQ-5D-5L index between the non-frailty and frailty groups was −0.14 (95%CI = −0.21; −0.06), indicating a 14% reduction of HRQOL compared to the maximum score (1.0). However, after adjusting for other covariates such as socioeconomic, behavioral, health status, and healthcare utilization characteristics, the estimated mean difference in the HRQOL between the non-frailty and frailty groups was −0.10 (95%CI= −0.17; −0.02) (R^2^ = 45.2%), showing a 10% reduction of the maximum EQ-5D-5L index. Meanwhile, the estimated mean difference in the EQ-5D-5L index between the non-frailty and pre-frailty groups was 0.02 (95%CI = −0.04; 0.08), but the difference was not statistically significant.

## 4. Discussion

Our study enriches the current literature about the prevalence of frailty in a low and middle-income country such as Vietnam, its associated factors, and the effects of frailty on HRQOL among older adults in the community. Our findings indicate that pre-frailty and frailty were common in Vietnamese community-dwelling older people. Moreover, frailty was associated with a significant reduction of HRQOL, and this relationship was preserved even being adjusted to a number of covariates. This study also highlighted some highly vulnerable people to frailty, suggesting potential implications for developing interventions to enhance older people’s health in the future.

In this study, one-fifth of our sample suffered from frailty, which was lower than the previous study in hospital settings in Vietnam (with 35.4% using Fried’s phenotype measure) [42]. It can be explained by the fact that hospitalized older patients might experience severe health problems, leading an increase in the risk of frailty [42]. Our prevalence could be comparable to the overall prevalence of frailty among older adults in the community (5.4–44%) in other developing countries, but much lower than the frailty rates in hospitalized older patients (27.8–71.3%) [47]. Our findings were higher than those of other studies in the rural community using similar instruments such as Mexico (8.6%) [48], Colombia (12.2%) [49], Turkey (7.1%) [50], or Africa (5.4–13.2%) [51]. The reasons for this diversity might be due to the difference in criteria or items used for assessing frailty in each study. Prior studies argued that the frailty rate in each community might vary, which depended on the definition, population, and settings [49,50,52]. Notably, our findings aligned with previous work that people having cognitive impairment, a history of multi-comorbidities, and falls were more vulnerable to frailty compared to those not having these conditions [3,10,27,42]. With such significantly high prevalence, it is suggested that older people in the community should be regularly screened for frailty to effectively prevent the adverse outcomes such as cardiovascular diseases, depression, fractures, or even deaths, which are consequences of frailty [2,3,4,5].

In line with previous studies, we found that people with a higher age and women were more likely to suffer from frailty [6,53]. Moreover, a widowed condition possibly increases the risk of having frailty by 73% among older people compared to those who have a spouse/partner. The death of a spouse/partner might be an extreme stressor that could be a harbinger of mental illness and thus elevate the vulnerability to frailty among widowed males [54,55]. Interestingly, our results showed that farmers had a lower likelihood of having frailty than that of retired people. Since the aging population is increasing rapidly in Vietnam, this finding suggests that vocational guidance and employment opportunities should be provided to the retired older adults, which could take advantage of their skills and knowledge as well as prevent the occurrence of frailty.

In the current study, we also examined the effects of frailty on HRQOL via the EQ-5D-5L index. After adjusting for covariates, we found that frailty contributed to a 10% reduction of HRQOL. Our results were higher than those of a study in Sri Lanka, which found that a 7.3% reduction of quality of life (QOL measured by the Older People’s Quality of Life Questionnaire) could be attributable to frailty conditions [27]. Another study in Italy using a similar instrument indicated that frailty reduced 6.4% of QOL in comparison to the non-frailty group [56]. These variances might be justified by the differences in study settings and HRQOL measures. However, this result partly underlines that frailty has a remarkably high influence on the HRQOL of older adults in rural communities in Vietnam. It should be noted that we observed a significantly high prevalence of having problems among frail people in each HRQOL dimension, especially pain/discomfort, mobility, and usual activities compared to non-frailty and pre-frail people. These findings were in line with a previous study in Taiwan, which showed that frail older people had a significantly lower score in the physical component (reduced approximately 6%) compared to the non-frailty group [13].

Our major limitations included the use of cross-sectional design, which did not allow us to conclude the causal relationship between frailty and its associated factors, as well as frailty and HRQOL reduction. A further longitudinal cohort should be warranted to examine our findings. Additionally, our questionnaire comprised a number of self-reported questions, which might result in recall bias. Our interviewers attempted to address this issue by explaining the questionnaire clearly, and used several recall techniques to help participants remember their answers. Furthermore, we could not find any previous studies using the EQ-5D-5L to examine the HRQOL of frail older people; thus, this limits our comparability to other settings. Finally, this study was only conducted in one rural district in northern Vietnam, which constrains our ability to generalize the study findings to other settings in Vietnam.

## 5. Conclusions

This study emphasized the high prevalence of frailty among older adults in the rural community of Vietnam. Frailty was also associated with a small reduction of HRQOL in this population. Interventions to prevent frailty and enhance HRQOL should include employment provision programs and routine screening, as well as pain, mobility, and daily activity management and assistance.

## Figures and Tables

**Table 1:** Demographic characteristics of respondents.

Characteristics	Non-Frailty		Pre-Frailty		Frailty		Total		*p*-value
	*n*	%	*n*	%	*n*	%	*n*	%	
Total	65	12.7	336	65.6	111	21.7	512	100.0	
Gender									
Male	14	9.1	109	70.8	31	20.1	154	30.1	0.184^a^
Female	51	14.2	227	63.4	80	22.4	358	69.9	
Age groups									
60–65 years	32	27.1	74	62.7	12	10.2	118	23.1	<0.01 ^a^
66–70 years	18	14.6	82	66.7	23	18.7	123	24.0	
71–75 years	10	10.0	72	72.0	18	18.0	100	19.5	
76–80 years	3	4.9	45	73.8	13	21.3	61	11.9	
>80 years	2	1.8	63	57.3	45	40.9	110	21.5	
Occupation									
Retired	16	7.4	143	66.2	57	26.4	216	42.2	<0.01 ^a^
Farmer	38	17.0	155	69.2	31	13.8	224	43.8	
Others	11	15.3	38	52.8	23	31.9	72	14.1	
Marital status									
Have spouse/partner	54	16.7	218	67.3	52	16.1	324	63.4	<0.01 ^a^
Single/divorced/separated	3	12.0	17	68.0	5	20.0	25	4.9	
Widowed	8	4.9	101	62.4	53	32.7	162	31.7	
Caregiver									
Spouse/children	61	13.6	298	66.5	89	19.9	448	87.5	0.04 ^a^
Grandchildren	2	5.3	21	55.3	15	39.5	38	7.4	
Others	2	7.7	17	65.4	7	26.9	26	5.1	
Smoking status									
Never	57	12.3	300	64.8	106	22.9	463	90.4	0.05 ^a^
Former smoker	1	4.6	18	81.8	3	13.6	22	4.3	
Current smoker	7	25.9	18	66.7	2	7.4	27	5.3	
Alcohol use									
Never	54	13.3	262	64.5	90	22.2	406	79.3	0.38 ^a^
Former alcohol user	3	21.4	7	50.0	4	28.6	14	2.7	
Current alcohol user	8	8.7	67	72.8	17	18.5	92	18.0	
	Mean	SD	Mean	SD	Mean	SD	Mean	SD	
Age	67.4	4.9	72.6	7.7	76.7	9.2	72.8	8.2	<0.01 ^b^

^a^ Chi-squared test; ^b^ Kruskal–Wallis test.

**Table 2:** Health status and health service utilization according to frailty status.

Characteristics	Non-Frailty		Pre-Frailty		Frailty		Total		*p*-value
	*n*	%	*n*	%	*n*	%	*n*	%	
Comorbidities									
Stroke	0	0.0	7	2.1	3	2.7	10	2.0	0.44 ^a^
Transient ischemic attack	2	3.1	22	6.6	3	2.7	27	5.3	0.20 ^a^
Diabetes mellitus	1	1.5	11	3.3	12	10.8	24	4.7	<0.01 ^a^
Chronic lung disease	1	1.5	23	6.9	9	8.1	33	6.5	0.20 ^a^
Parkinson’s	0	0.0	2	0.6	0	0.0	2	0.4	0.59 ^a^
Arthritis	22	33.9	85	25.3	37	33.3	144	28.1	0.14 ^a^
Hypertension	13	20.0	104	31.0	49	44.1	166	32.4	<0.01 ^a^
Number of comorbidities									
0	32	49.2	156	46.4	33	29.7	221	43.2	<0.01 ^a^
1	28	43.1	115	34.2	51	46.0	194	37.9	
≥2	5	7.7	65	19.4	27	24.3	97	19.0	
History of fall in the last 12 months	12	18.5	63	18.8	34	30.6	109	21.3	0.03 ^a^
Cognitive impairment (Mini cog)	26	40.0	225	67.0	89	80.2	340	66.4	<0.01 ^a^
Polypharmacy (≥5 drugs)	3	4.6	45	13.4	24	21.6	72	14.1	<0.01 ^a^
Using inpatient service in the last 12 months	7	10.8	53	15.8	18	16.2	78	15.2	0.56 ^a^
Using outpatient service in the last 12 months	30	46.2	166	49.4	57	51.4	253	49.4	0.80 ^a^
Regular health examination in the last 12 months	44	67.7	240	71.4	83	74.8	367	71.7	0.59 ^a^

^a^ Chi-squared test; percentages are relative to the total number of people for a particular group.

**Table 3:** logistic regression models to identify associated factors with having frailty. OR: odds ratio.

Characteristics	Adjusted OR	*p*-value	95%CI	
Age	1.05	<0.01	1.02	1.08
Marital status				
Have spouse/partner	ref			
Single/divorced/separated	0.71	0.56	0.23	2.23
Widowed	1.73	0.03	1.06	2.83
Current occupation				
Retired	ref			
Farmer	0.54	0.03	0.32	0.93
Others	1.25	0.49	0.66	2.39
Cognitive impairment (Mini cog)				
No	ref			
Yes	1.88	0.02	1.09	3.25
Number of comorbidities				
0	ref			
1	2.33	<0.01	1.38	3.94
≥2	1.97	0.03	1.06	3.67
History of fall in the last 12 months				
No	ref			
Yes	1.91	0.02	1.13	3.24

**Table 4:** Percentage of patients having problems in each domain of EuroQol-5 Dimensions-5 Levels(EQ-5D-5L)according to frailty status.

Characteristics	Non-Frailty		Pre-Frailty		Frailty		Total		*p*-value
	*n*	%	*n*	%	*n*	%	*n*	%	
Having problems in mobility									
No	42	64.6	186	55.4	28	25.2	256	50.0	<0.01 ^a^
Yes	23	35.4	150	44.6	83	74.8	256	50.0	
Having problems in self-care									
No	50	76.9	272	81.0	64	57.7	386	75.4	<0.01 ^a^
Yes	15	23.1	64	19.0	47	42.3	126	24.6	
Having problems in usual activity									
No	47	72.3	199	59.2	39	35.1	285	55.7	<0.01 ^a^
Yes	18	27.7	137	40.8	72	64.9	227	44.3	
Having problems in pain/discomfort									
No	15	23.1	105	31.3	21	18.9	141	27.5	0.03 ^a^
Yes	50	76.9	231	68.8	90	81.1	371	72.5	
Having problems in anxiety/depression									
No	40	61.5	231	68.8	53	47.8	324	63.3	<0.01 ^a^
Yes	25	38.5	105	31.3	58	52.3	188	36.7	
	Mean	SD	Mean	SD	Mean	SD	Mean	SD	
EQ-5D-5L index	0.70	0.18	0.70	0.19	0.58	0.20	0.68	0.20	<0.01 ^b^

^a^ Chi-squared test; ^b^ Kruskal–Wallis test; SD: standard deviation

**Table 5:** regression models to identify the association between frailty status and health-related quality of life(HRQOL).

Models	EQ-5D-5L Index		
	Coefficient (95%CI)		
	Pre-Frailty	Frailty	R^2^ (%)
Model 1: Unadjusted (Univariate regression model)	0.01 (−0.05; 0.07)	−0.14 (−0.21; −0.06)	14.6
Model 2: Model 1 + age + sex	0.01 (−0.05; 0.07)	−0.13 (-0.20; −0.05)	20.6
Model 3: Model 2 + occupation	0.01 (−0.05; 0.07)	−0.12 (–0.20; −0.05)	21.4
Model 4: Model 3 + marital status + caregiver	0.01 (−0.05; 0.07)	−0.12 (−0.20; -0.05)	21.4
Model 5: Model 4 + smoking + alcohol	0.00 (−0.06; 0.06)	−0.13 (−0.21; -0.05)	26.9
Model 6: Model 5 + number of suffered morbidities + cognitive impairment + fall in the last 12 months	0.02 (−0.04; 0.08)	−0.09 (−0.17; −0.02)	38.5
Model 7: Model 6 + polydrug use + inpatient use + outpatient use + regular health examination	0.02 (−0.04; 0.08)	−0.10 (−0.17; −0.02)	45.3

Coefficient indicates the difference in EQ-5D-5L index between pre-frailty and non-frailty, and between frailty and non-frailty groups.
